# A Model for Effective Nonverbal Communication between Nurses and Older Patients: A Grounded Theory Inquiry

**DOI:** 10.3390/healthcare10112119

**Published:** 2022-10-22

**Authors:** Esther L. Wanko Keutchafo, Jane Kerr, Olivia B. Baloyi

**Affiliations:** School of Nursing and Public Health, College of Health Sciences, University of KwaZulu-Natal, Durban 4001, South Africa

**Keywords:** effective nonverbal communication, nurse–older patient relationships, grounded theory, Cameroon

## Abstract

Nonverbal communication is an inevitable art to be effectively mastered by nurses. Nurse nonverbal communication has many benefits when it is effective. For instance, nonverbal communication is important to convey affective and emotional information, and demonstrate respect for and build therapeutic relationships with older patients. As the older population is growing fast worldwide, effective nonverbal communication with older patients is an essential skill for nurses and will improve patients’ satisfaction and the quality of care. Therefore, this article presents a model to guide effective nonverbal communication between nurses and older patients. A Grounded Theory approach guided the study. Data were collected between July 2018 and January 2020 through overt participant observations and individual interviews. Purposive and theoretical sampling were used to select 13 clinically experienced nurses, 4 nursing students, and 8 older adults. Data analysis encompassed open coding, axial coding, and selective coding. The results showed that effective nonverbal communication emerged as the co-phenomenon hinged within context and/or environment and is influenced by certain factors. This model, which is in support of person-centered communication and care, advocates for effective nonverbal communication between nurses and older patients.

## 1. Introduction

Worldwide, older adults account for 1.05 billion people, with 74.4 million in African countries with the expectation to reach 235.1 million by 2050 [1]. In sub-Saharan Africa, the burden of geriatric diseases is growing, with more older adults requiring geriatric healthcare services and frequent hospitalization with longer stays [2,3]. This is especially the case in sub-Saharan Africa where there are few long-term care settings [4]. With hearing deficits, changes in attention and coding of information, and restrictions in interaction, participation, and effective verbal communication [5], nurses’ effective communication with older adults emerges as an essential skill in geriatric care [6].

Communication, which is important to understand older adults’ needs and support their health and well-being [7], is defined as the process of sending and receiving messages to share knowledge, attitudes, and skills [8]. It includes both verbal and nonverbal components, since it is not just the mere transmission of information [9]. While verbal communication denotes the transmission of messages through spoken words [8], nonverbal communication describes the reaction of the face, body, or voice, including what is expressed between each other [10,11]. Nonverbal communication is important to convey affective and emotional information, demonstrate respect for patients, and build therapeutic relationships with patients [12,13]. This makes nonverbal communication unique and more important for effective communication between nurses and older patients. When nurses enhance their communication skills, it improves patients’ satisfaction as well as the quality of care [14,15].

Communication, as one of the important aspects of caring for patients that affects all other aspects of care, should be given special attention [16]. To date, there are few nonverbal communication models identified to help nurses to communicate effectively with patients let alone older adults. The first model is SOLER (Square, Open, Lean, Eye contact, Relax) developed in 1975 by Eagan to describe effective body language employed to make others feel listened to. It only includes proxemics (use of space) and kinesics (movements of the body), and mostly focuses on interactions during a consultation, not during hospitalization [17]. The second model is SURETY (Sit at an angle, Uncross legs and arms, Relax, Eye contact, Touch, Your intuition), which criticizes and advances the SOLER model by including the use of touch, emphasizing the importance of individual intuition, and encouraging the inclusion of therapeutic space [18]. Although it includes proxemics, kinesics, and haptics (use of touch), it has been developed to encourage the inclusion of therapeutic space and intuition in verbal communication skills’ content. None of these models were intended for nurses’ effective nonverbal communication with older patients or were derived from the participants’ views on nonverbal communication between patients and nurses. Moreover, a model with consideration of nurses’ views is more likely to be appropriate and acceptable by nurses [19], because healthcare workers’ perspectives are important in determining effective strategies [20].

As of 2018 in Cameroon, the growing older population is translating to increased healthcare demand [2]. Unlike other African countries such as Mauritius, Seychelles, and South Africa, there is no national effort to develop long-term care settings in Cameroon [4]. As a result, older adults solely utilize hospital settings when requiring medical assistance [21,22] where nurses communicate more often with them. Additionally, Cameroon is one of the most linguistically fragmented countries in sub-Saharan Africa, with approximately 250 indigenous languages, apart from English and French which are both considered official languages [23]. As a result, it is less likely that a nurse speaks the same vernacular as an older patient who does not speak French or English. Although communication skills training and models do not necessarily ascertain that nurses will be skilled communicators [24], they might be helpful in assisting nurses to improve their nonverbal communication with the older adult population. As some of these older adults mostly rely on nonverbal communication because of their functional impairments [25], nurses need to be equipped, more than ever, with tools to improve their communication skills. Therefore, this paper aims to present a model for effective nonverbal communication between nurses and older patients.

## 2. Materials and Methods

### 2.1. Design

The purpose of this study was to develop a model for effective nonverbal communication between nurses and older patients during hospitalization. It was for this reason that a qualitative Grounded Theory (GT) approach was followed [26]. GT was chosen because “it is a useful methodology for the study of interpersonal activities between nurses and patients and others because a social interaction is at the heart of the caring process in nursing” [27] (p. 16).

### 2.2. Study Settings and Context

The study took place in two public hospitals in the east and central regions of Cameroon, a low-and-middle-income country at the heart of the Gulf of Guinea in Central Africa [28]. Both hospitals are in the central level of the three-level pyramidal Cameroonian healthcare system. The first hospital was chosen because it is the only one with a geriatric unit in Cameroon. Similarly, the second was selected because it is a referral regional hospital. In both hospitals, older adults are admitted to adult wards with younger adults but are categorized according to their illness. In addition, both hospitals employ qualified nurses and nurse assistants, irrespective of registration status given that registration was not mandatory in Cameroon before 2022.

### 2.3. Study Participants and Sampling Methods

In keeping with GT, which aims to recruit participants with rich information on the phenomenon under investigation, purposive and later theoretical sampling were used. Firstly, 10 clinically experienced nurses who were involved in the day-to-day care of older adults admitted to the hospital, could articulate in English or French, and were willing to participate in the study were purposively sampled. Furthermore, 8 older adults who were not critically ill, could articulate in English or French, and expressed a willingness to participate in the study were also purposively sampled. Theoretical sampling included the recruitment of additional participants who cared for older patients. These were 2 middle unit managers, 4 undergraduate student nurses allocated for clinical placement in the selected hospitals, and 1 nurse assistant. We collected and analyzed data simultaneously as recommended in Grounded Theory; thereafter, we stopped recruiting and including participants when no additional information emerged from the analysis. All up, 17 nurses and 8 older patients were included in the study. Their characteristics are described in Table 1.

### 2.4. Data Collection

Data were collected between July 2018 and January 2020 through overt participant observations and individual interviews. The principal investigator commenced with a month-long observation of how nurses communicated nonverbally with older patients during different types of interactions. Such interactions were, but are not limited to, day-to-day nursing care-related tasks, social interactions, and health education interactions. The observations were recorded as field notes because no ethics permission was granted for video recording. Data from the observations guided the development of the initial interview guide which was used to conduct individual in-depth interviews with participants. 

Only the nurses who were observed met the criteria to be interviewed. Therefore, the principal investigator approached them in the nursing station when they seemed free, verbally provided information about the study, and issued each with an information letter with the intention to obtain consent to be interviewed. Following this, those nurses who showed interest in participating in the study were booked for individual in-depth interview at times most convenient for them. The initial interviews captured the meaning and channels of nonverbal communication from the nurses’ perspectives. One open-ended question was asked: “How can you define nonverbal communication with older patients”? This was followed by probing questions that allowed the researcher to elicit more information, obtain more clarity, and confirm data captured during observations. Due to the constant comparative methods for data collection and analysis, interviews informed each other. Each individual interview, conducted in the participant’s preferred language, lasted between 50 min and 60 min. Subsequently, field notes were recorded during and after the interviews. Data saturation was achieved at interview 17 when no additional information emerged. 

The same principles were followed to recruit and include older adults for interviews. A total of 47 older adults were referred to the study, but 13 did not meet the eligibility criteria. The remaining 34 older patients who met the inclusion criteria were individually approached at their bedside when they seemed free with no visitors nor care activities happening. The principal investigator introduced herself, explained the purpose of the study, and sought consent for participation in the study. Consent to be observed was provided by 29 older patients, of whom 8 were interviewed thereafter. Older adults who consented to participate in the study either agreed to be interviewed on the spot or preferred to make an appointment for a different time. The initial interviews with older adults captured their interpretation and understanding of nurse nonverbal communication. One open-ended question was asked: “How do you understand when a nurse communicates without saying a word?” This was also followed by probing questions for more clarity and to obtain additional information. The interviews with older adults also informed each other. Field notes were also taken during and after interviews. Data saturation was achieved at interview 8 when no additional information emerged. 

### 2.5. Data Analysis

Data were analyzed by three researchers, who were all female and comprised a principal investigator and two academics with PhDs who have supervised graduate students following qualitative research methodology. None of the researchers worked or was working at the data collection site; therefore, they had no relationship with the participants. Data analysis encompassed open coding, axial coding, and selective coding, which seemed intertwined as the researchers moved back and forth between data collection and data analysis. The process is referred to as a constant comparative method by Strauss and Corbin [29]. This allowed the generation of increasingly focused questions, thus providing direction for subsequent interviews [30]. In addition, constant comparison was used throughout the study. The software NVIVO version 12 [31] was used to import transcripts, write memos, code conceptual categories, properties, and dimensions from the data, conduct data analysis, and refine the model. 

Data were initially coded sentence-by-sentence during open coding to summarize and define emerging categories, paying special attention to the processes linking them. This was followed by axial coding, where data were reassembled and codes refined and categorized into categories and subcategories [32]. This allowed for a better understanding of the categories, with similar ones merged into higher-order categories. After creating concepts and categories from data in the open coding phase, the researchers continued to group categories and subcategories in the axial coding phase. The researchers then developed a category by specific conditions, context, and actions or interactions by which it was managed [33]. The researchers further refined a list of categories by carefully trying to merge or delete some of them after making possible connections. Categories were linked depending on their properties and dimensions. Some categories were named in words and phrased by the participants, while others were renamed by the researchers’ academic and professional knowledge and readings. These concepts are referred to as “literature-driven concepts” [29]. The researchers continued to code new data, re-examining and comparing the data until saturation was reached. Selective coding followed axial coding, which involved the process of selecting the core category “*effective nonverbal communication*”, systematically relating it to other categories, validating those relationships, and completing categories that needed further refinement and development; by following the process of reduction and comparison. The iterative nature of the data analysis process allowed the researchers to repeatedly ask questions while studying the data, in addition to using the “*waving a red flag*” *technique*, which allowed them to look beyond the obvious in the data [26]. The researchers were convinced that the model began to emerge as soon as the diverse properties began to integrate. 

### 2.6. Ethical Considerations

Ethical approval (reference number HSS/2008/017D) to commence the research study was obtained from the University of KwaZulu-Natal Humanities and Social Sciences Research Ethics Committee. Permission was further obtained from the two participating hospitals. Following ethical approval requirements, before data collection, an information letter explaining the purpose and nature of the study was given to each participant. The participants were allowed to ask any questions before the voluntary signing to participate in the study, be observed, and be audio-recorded. The participants were informed that they may withdraw from the study at any time with no due penalty or repercussions. Furthermore, all participants were assured that no information provided by them would be shared with another person without their authorization. To maintain confidentiality, pseudonyms were used. Participants did not receive monetary benefits for participating in the study. 

### 2.7. Rigor

To ensure trustworthiness, the researchers used the criteria of credibility, transferability, dependability, and confirmability [34]. The credibility of the study was promoted by the researchers’ prior engagement with participants. Prolonged engagement was ensured by the establishment of relationships with participants during the study. Data analysis was audited by taking observational field notes regarding the context of the interviews, with peer debriefing conducted to confirm emerging categories and themes. Confirmability was ensured by triangulating data sources and validating audiotaped and transcribed transcripts against emerging categories and themes through constant comparison. Further, nine interviews were returned to participants who did not add much to what they originally said. Dependability was ensured by data quality checks with an expert in Grounded Theory, peer review of coding, and consultation with qualitative researchers to validate the codes and categories that emerged from the analysis. Finally, transferability was established by rich descriptions of the study context, informants, research procedures, and the provision of extracts from the interviews to enrich the findings.

## 3. Results and Discussion

The model was developed based on the findings from open coding, selective coding, and axial coding. Table 2, Table 3 and Table 4 summarize the extracts from the participants and the observations, which served as a starting point to develop the model. 

**Figure 1:**
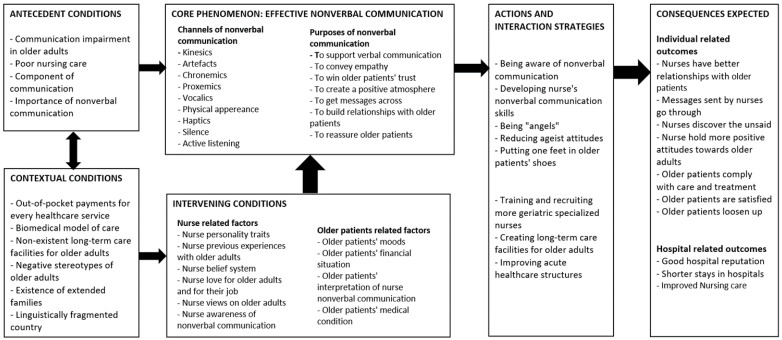
Summary of findings in line with Strauss and Corbin’s paradigm.

Additionally, Figure 1 indicates the elements of the model in line with Strauss and Corbin’s paradigm, which include the antecedents, the contextual conditions, the core phenomenon, the actions and interaction strategies, the intervening conditions, and the outcomes. 

These elements (Figure 1) were used as the foundation for the development of this model. Some of these elements were extensively described in other papers by the same lead author [35,36]. Hence, this paper focuses on the emerged model, to enhance nonverbal communication between nurses and hospitalized older adults. 

We followed the components for developing a model, which include the purposes of the model, the concepts and their definitions, the structure of the model, and the assumptions of the model, as described by Chinn and Kramer [37].

### 3.1. Purpose of the Model

According to Chinn and Kramer [37], the purpose of the model justifies the context and situation in which the model applies. Although communication is bidirectional, nurses are responsible for its proper conduct [38]. Therefore, this model of effective nonverbal communication between nurses and older patients, in the context of this study, provides a framework that guides nurses to effectively communicate nonverbally with older adults in hospital settings. Furthermore, in-service training for nurses who were not part of this study can be developed based on the elements provided by this model. This model can be used by curriculum developers and policymakers as a guide for nursing schools in the teaching and learning of nonverbal communication to both undergraduate and postgraduate students. Furthermore, this model answers the United Nations’ [39] call for more data on older adults from developing countries, thus contributing to the limited body of knowledge in the area of nonverbal communication in geriatric care in hospital settings [40], as compared to nonverbal communication in long-term care settings. 

### 3.2. Basic Assumptions of the Model

The assumptions that formed the basis of effective nonverbal communication between nurses and older patients in this model are outlined below:

*Effective nonverbal communication is present* in every healthcare encounter between nurses and older patients because it is impossible not to communicate nonverbally [10]. In other words, whenever there is an interaction between a nurse and an older patient, nonverbal communication is inevitable even when there is no verbal content. Scholars have estimated the amount of nonverbal content in communication, in comparison to verbal content. They described that nonverbal communication accounts for 60% to 90% of total communication [13]. Thus, nonverbal communication is unavoidable. Therefore, nurses should be aware that their nonverbal communication might send conflicting messages to older patients if they do not match the verbal content. In addition, the awareness of nonverbal messages sent to others is essential, as it often provides an explanation as to why people respond to us in the way they do [41]. Hence, nonverbal communication emerges as an intentional concept, which nurses should be aware of, as it may have negative consequences to the level of care rendered. 

*Effective nonverbal communication with older patients is person-centered*. It is worth noting that older patients are not a homogenous group, as they have different experiences [42] coupled with different nonverbal communication needs. Person-centered care assumes that healthcare workers should communicate and interact with patients in a person-centered way while paying attention to patients’ different expectations and needs through verbal and nonverbal communication [43]. Hence, an added assumption in this model is that nonverbal communication is individualized and needs-oriented. Nurses are encouraged to take into consideration older patients’ nonverbal communication needs. Despite this, authors acknowledge the beliefs of Chan et al. that initial interactions with older patients tend to be scripted and governed by established social norms [44]. In time, nurses should be able to easily bend or break these norms to align them with each older patient’s specific needs. 

*Effective nonverbal communication is unique, dependent on the context and the nurse rendering care*. The model brings forth the assumption that clinical contexts are different, along with the types of interaction with patients and the types of illnesses. On the other hand, nurses bring to the table different backgrounds, training, and personalities. These lead to unique encounters with each one. The emphasis in this model is that unique does not mean chaotic but instead means distinct, that may or may not be automatically replicable to another encounter. Moreover, effective nonverbal communication cannot be reduced to a set of theoretical and linear principles to absolutely follow because there is no universal way to communicate. This allows room for the creativity, flexibility, intuition, and authenticity that are needed in effective communication [44]. Furthermore, as nurses grow in confidence and experience, the model assumes that they will embrace and master effective nonverbal communication in every encounter and obtain mastery over the external display of their emotions. Hence, nurses will become shapers of and accountable for effective nonverbal communication with older patients. 

*Effective nonverbal communication is a subjective and interactive process* which may be misinterpreted or misunderstood. Indeed, there is a risk of miscommunication or misunderstanding that cannot be eliminated when using nonverbal communication [45]. In this model, we posit that nurses interpret situations based on filters and frames. Filters refer to what influences the way nurses attempt to communicate nonverbally with older patients. Such filters are, but are not limited to, nurses beliefs, past experiences, and personality traits [36]. On the other hand, frames can be defined as a nurse’s own interpretation of a situation. As an example, one participant reported that some older patients practice witchcraft in the hospital, therefore preventing nurses from getting closer to them or spending more time with them. According to the participant, this may have negative consequences on the effectiveness of nonverbal communication between nurses and older adults. As nonverbal communication is an interactive process, nurses may misunderstand and misinterpret nonverbal messages sent by older patients. Like nurses, older patients can misunderstand or misinterpret the nonverbal messages sent to them, resulting in ineffective nonverbal communication. The mismatch in the interpretation and understanding of nonverbal communication may be due to past negative experiences with nurses, critical conditions, or different cultures or religions between nurses and older adults [36]. To minimize misinterpretations and misunderstanding, the model suggests that nurses be encouraged to obtain feedback that ascertains that the older patients have understood, or not, the nonverbal messages sent by nurses. Similarly, nurses should ascertain that they have correctly captured messages sent to them by older patients for the success of nonverbal communication. This is called reaching an area of communicative communality [46].

*Effective nonverbal communication is reliant on cultural and religious beliefs* complicated by the multilingual nature of the context. Hence, the assumption in this model is that within effective nonverbal communication are the components of religion and culture. As an example, in some cultures or religions, eye contact with an older adult is considered rude; conversely, it can express empathy in other contexts. Another example is affective touch, which can be considered invasive in some contexts. Hence, the model posits that effective nonverbal communication is reliant on one’s culture and religion. Within the context of this study, nurses and older patients are often from religious and culturally diverse regions with language differences. Cameroon is known for being multilingual with more than 250 indigenous languages [23] in a population of more than 26 million people. Although there may be instances where both nurses and patients share the same cultural and religious beliefs, the assumption in this model is that different social circumstances, orientations, and languages may influence nonverbal communication. Nurse prudence is therefore essential when initiating nonverbal modalities that can be considered ambiguous. 

### 3.3. Concepts and Definitions

Effective nonverbal communication is the core concept from which other concepts evolve. It is a dynamic and evolving process that takes place as the relationship with an older patient develops. The emerging concepts in this study and those described in this paper are effective nonverbal communication, context and environment, action and interaction strategies, pillars, and outcomes.

#### 3.3.1. Core Concept

The core concept in this study is effective nonverbal communication between nurses and older patients. It refers to a variety of communicative behaviors that do not carry linguistic content, but are unique, religiously and culturally sensitive, and person-centered. In the literature, common attributes of effective communication include a significant tool in planning and implementing person-centered care, a foundation for interpersonal relationships, and a determinant of promoting respect and dignity [47,48,49]. On the other hand, inaccurate or ineffective nonverbal communication behavior will not enable older patients to understand and interpret nurse messages. Therefore, it should be accurate to avoid distortion of messages. In this model, effective nonverbal communication entails the channels and the purposes of nonverbal communication in the context of the study. However, the core concept has been extensively discussed in another manuscript [50]. Therefore, the following is a summary of the core concept.

##### The Channels of Effective Nonverbal Communication

The channels of effective nonverbal communication mostly include haptics, proxemics, kinesics, and vocalics. Few participants mentioned active listening, physical appearance, and artefacts. 

*Haptics* refer to the use of touch or physical contact, which in this study includes handshake, kiss, hug, pat, and stroke. 

*Proxemics*, the use of space and distance, are the physical proximity and distance with older patients. In this model, physical proximity refers to sitting close to older patients, including sitting on their beds. Physical proximity includes standing at the door to talk to them, sitting far from them, and having their back towards them. 

*Kinesics* are the movements of any part of the body, such as smiling, frowning, leaning forward, and waving hands. 

*Vocalics* are the aspects of the voice used when communicating with older patients. In this study, speaking too loudly, too fast, or even too slow were reported by participants. 

*Artefacts* refer to the use of objects during communication. In this study, some participants reported that they show a bottle or the medication to some older patients who did not understand French to express the time to drink medication. It was followed by a change of position by the older patient, showing that he has understood the message and was ready to swallow his tablets. 

*Physical appearance* refers to how nurses dress when they come to work. As described by one participant in this study, a nurse with a uniform can still look like a drug addict. Another one said that a nurse with a see-through uniform could sexually provoke older male patients. 

##### The Purposes of Effective Nonverbal Communication

The purposes of effective nonverbal communication: the ultimate purpose of nonverbal communication is to help patients with their coping and recovery during hospitalization [51]. In this study, nurses reported that nonverbal communication assisted them in building relationships with older patients, winning their trust, creating a positive atmosphere, supporting verbal communication, reassuring, and conveying empathy to older patients. 

*To build relationships*: Effective nurse–patient communication has been proven to be fundamental to building a positive relationship between nurses and patients [52]. Hence, this model advocates for nurses to use one or more channels of nonverbal communication to express their willingness to build relationships with older patients. 

*To win patients’ trust*: Kourkouta and Papathanisou recommend that for nurses to develop relationships with their patients, they must be mindful of their first encounter with those patients because first impressions last forever [35]. Therefore, we encourage nurses to be aware of their body language on their first encounter with older adults. 

*To support verbal communication*: Communication has two components, namely, verbal and nonverbal. The differences in the native languages of nurses and patients creates communication barriers [53]. Moreover, verbal communication and nonverbal communication can conflict with each other in one interaction [10] and patients believe the nonverbal when verbal communication is incongruent with nonverbal communication [54]. Therefore, this model encourages nurses to ensure the congruency of both verbal and nonverbal communication. 

*To create a positive atmosphere*: The hospital environment is stressful to older patients. The noise of machines, the unfamiliar healthcare workers and environment, the pain, the discomfort, and the uncertainty of death lead to patients’ emotional fluctuations [55] in an atmosphere of fear and anxiety. Therefore, nurses are encouraged to use nonverbal communication to create a positive atmosphere or to change a negative atmosphere into a positive one. 

*To convey empathy*: Empathy is the ability to understand and share another person’s emotions [56]. Nurses are encouraged to communicate to older patients that they are compassionate, interested, and concerned about their situations. Knowing the changes that older adults undergo concerning their physical, psychological, social, and environmental health will help nurses better understand older patients [57].

#### 3.3.2. Context and Environment

Anderson and Risor [58] have argued about the importance of contextualization and how it relates to the notion of causality for eventual understanding and insight. In this study, the *context* refers to the types of encounters between nurses and older patients. These range from encounters around health communication, nursing tasks, activities of daily living, and normal social life, as described by Barker et al. [59]. The context also encompasses the nursing shortages, excessive workload, and poor communication skills that have been identified by Kwame and Petrucka as some barriers to effective communication with patients [60]. Wards in Cameroon have limited resources and there are out-of-pocket payments for every healthcare service. For example, if patients cannot afford to pay for cotton wool or syringes, they will not receive their prescribed injections. Ward staffing is often limited to one staff member per shift, which limits the interaction of the nurse with the older adult due to lack of time versus accomplishment of the routine. 

*The environment*, within this model, is the ward and the persons involved in the communicative encounter, namely, the nurses, the older patient, and/or the relatives. The ward is mostly a medical ward because there are very few geriatric units in acute settings in Cameroon. Similar to Cameroon, in Ghana [57], older adults are mostly nursed in general wards together with young and middle-aged adults after diagnosis has been classified as a medical or surgical case. In the wards, at least one relative is requested to stay with the older patient 24/7. During their stay, the relatives participate to care (personal hygiene, medicine intake, temperature checking, etc.) when nursing teams are short-staffed and/or alert the nurses when problems arise, such as in Malawi [61]. Moreover, the presence of relatives in the ward has been reported as a nuisance to care [62,63]. All employed nurses are certified but not necessarily registered with the Nursing Council, as registration was not compulsory for practice before 2022. Some older adults are often seen as witches by the community and the healthcare population, similar to Ghana [57] and Uganda [64]. On the other hand, some are also seen as babies or as intelligent people. All the above-mentioned constitute the context and the environment for effective nonverbal communication between nurses and older patients.

#### 3.3.3. The Action and Interaction Strategies

To achieve effective nonverbal communication with older patients, participants reported on a series of strategies that needed to be put in place, referred to as action and interaction strategies according to the GT language. These were, but are not limited to, being aware of one’s nonverbal communication, being “angels”, putting yourself in the shoes of older patients, and reducing negative attitudes towards older patients. Additionally, creating long-term care facilities, improving acute healthcare structures, enhancing communication skills through education and training, and recruiting more gerontologist nurses were mentioned as strategies for effective nonverbal communication with older adults. However, they will not be discussed in this paper.

*Awareness of nonverbal communication*: Nonverbal messages are often subconsciously transmitted; thus, nurses tend to be neither aware nor mindful of the value of nonverbal communication when communicating with older patients. In this study, some nurses reported that they had never used nonverbal communication with older patients. This means that they were not aware that they have been using nonverbal communication. Moreover, awareness of one’s nonverbal messages leads to a greater understanding of the messages exchanged [65]. Nurses should be on constant guard of their NVC to ensure maximum satisfaction of patients [66], especially their kinesics and proxemics [67]. After all, awareness of nonverbal communication explains why people respond to us the way they do, and influences how the other person communicates with us [41]. This means that if older patients respond to nurses in a certain way, it is because of nurses’ nonverbal communication.

*Being “angels”:* Participants described that to achieve effective nonverbal communication with older patients, nurses should be “angels”. Angels are commonly described as spiritual beings who do good. In this study, being an angel entailed showing concern and interest in older adults, being kind and close to older adults, and conveying empathy. Furthermore, the angelic being of nurses is further evident in their soft voice tones versus commanding tones and positive facial expressions. 

*Putting yourself in the shoes of older patients*: Ageing is an inevitable event, and it will happen to everyone in the absence of premature death. Nurses reported that they do imagine themselves as older adults. Therefore, they attempt to render imaginary care and nonverbal communication that they would want to receive if they themselves were hospitalized. This particular study finding concurs with that of Van Der Cingel, who reported that nurses who cared for older people with a chronic disease put themselves in the patients’ shoes [68]. 

*Reducing negative attitudes toward older patients*: Ageist attitudes, which comprise discrimination, prejudice, and stereotypes toward a person based on their age, have been recognized as a factor influencing older adults [69,70]. Ageist attitudes can lead to age-based disparities in diagnostic procedures, decision-making, and types of treatment offered. As previously indicated, in this current study, some nurses avoided older patients because of alleged witchcraft. Additionally, some nurses shouted at older patients because they saw them as children. Moreover, ageist attitudes are reflected in interpersonal interactions that are patronizing or involve elder speak [71]. Ageism in healthcare limits older adults’ access to appropriate and respectful care, and results in adverse clinical outcomes [72]. Ageist attitudes are easy to deal with because although they are social constructs historically and culturally situated, they are individually interpreted [73]. Therefore, this model advocates for nonverbal communication free of age-related bias, which is essential to high-quality, patient-centered care. 

#### 3.3.4. The Pillars to Sustain Effective Nonverbal Communication between Nurses and Older Patients

For this model, pillars refer to factors that influence effective nonverbal communication between nurses and older patients. In this paper, we only list the pillars because they have been extensively discussed in Keutchafo and Kerr [35] and Keutchafo et al. [36]. The factors that influence effective nonverbal communication in this model are summarized as nurse-related and older-patient-related factors. The nurse-related factors are awareness of nonverbal communication, personality traits, previous experience with older adults, beliefs system, love for the job and for older patients, and views on older adults. The older-patient-related factors include moods, financial situation, interpretation of nurses’ nonverbal communication, and medical condition. 

#### 3.3.5. The Outcomes of the Model

This study evidenced that when nonverbal communication between nurses and older patients is effective, it yields positive outcomes. For this model, the outcomes are categorized as nurse-related, older-patient-related, and operational.

##### Individual-Related Outcomes

In this paper, we only describe the most cited outcomes by participants. They include better relationships between nurses and older patients, compliance with care and treatment, discovery of the unsaid, and older patient satisfaction. 

Communication encompasses the verbal, the nonverbal, and any form of interaction in which messages are created and meanings are derived to influence the nurse–patient relationship [60]. Likewise, in this model, it emerges that the outcome of effective nonverbal communication is *better relationships between nurses and older patients*. Although nurses and older patients are strangers at the beginning of the relationship, they are expected to improve their relationship through positive nonverbal communication. Participants in this study reported that they avoided nurses who were always shouting. Consequently, older patients will become closer to nurses who display positive nonverbal communication; this will lead to the betterment of their relationships.

**Figure 2:**
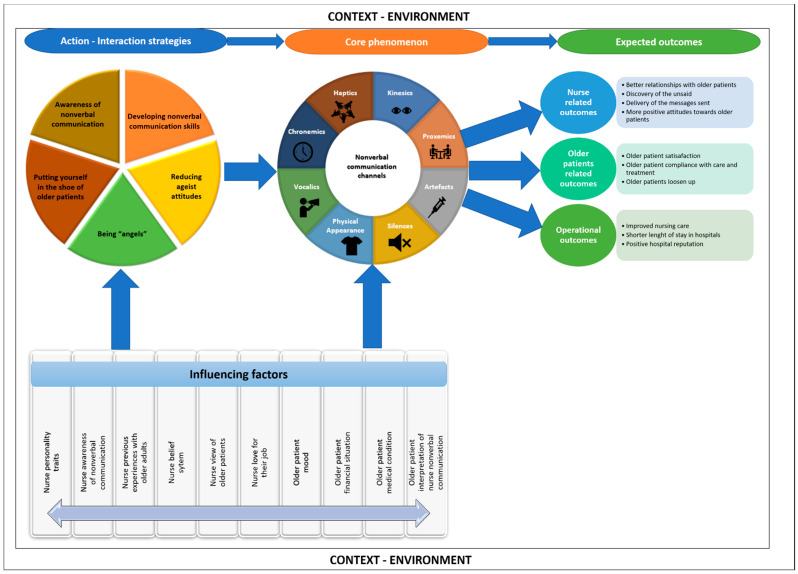
A model for effective nonverbal communication with older patients.

Sumijati et al. have argued that the essence of communication is relationships that can lead to changes in attitudes and behaviors [74], which in this model is referred to as *compliance with care and treatment*. One of the outcomes of effective nonverbal communication with older patients is compliance with care and treatment, as described in Figure 2. 

Nurses in this study reported that older patients did not want to take their medication nor accept certain care. Moreover, studies have shown that effective communication with patients leads to compliance to care and treatment [60]. As proposed in this model, older patients will be able to accept the care and treatment provided by nurses when nonverbal communication is effective.

The betterment of relationships is expected to lead to the discovery of what older patients do not express or have wrongly expressed. It has been shown that effective communication empowers patients to disclose their concerns and expectations [75], whereas patients would be less motivated to disclose their needs and feelings to nurses when they have past negative experiences in their interactions with nurses [76]. Moreover, patients need encouragement to talk about their psychological issues [77]. However, when communication is effective, older adults feel cared for, respected, and more able to describe their concerns [42]. This means that when relationships are better because of positive experiences in nonverbal communication with nurses, *nurses would discover the unsaid*. This is another important outcome in this model. 

*Older patient satisfaction* is one of the outcomes of effective nonverbal communication. Evidence shows that nurse nonverbal positive behaviors lead to higher patient satisfaction [78]. To improve patient satisfaction, nurses are encouraged to enhance their communication skills [63]. In this model, and as confirmed by Junaid et al., to ensure maximum satisfaction of patients, nurses should be on constant lookout of their nonverbal communication [66]. Such a level of awareness will prevent nurses from sending conflicting messages to older adults through their nonverbal communication. 

##### Operational Outcomes

*Improved nursing care* is one of the hospital-related outcomes. As confirmed by Tran et al., enhancing the effectiveness of verbal and nonverbal communication can improve the quality of care [14]. Effective nonverbal communication with older patients will make room for nurses to shift from task-oriented care to person-centered care. This will improve the quality of care rendered. 

When nursing care is improved, older patients will have *shorter lengths of stay in hospital*. Participants mentioned the reduction of length of stay in hospital because they viewed older patients as people who not only want to stay at home, but who also want to return home after hospitalization [79]. Moreover, studies support both a shorter or longer length of stay associated with better quality of care [80]. As nurses do not decide on the discharge or otherwise of patients, they are encouraged to use effective nonverbal communication with older patients irrespective of the length of stay.

Improved quality of care and shorter stays in hospitals will lead to a *positive reputation* for these healthcare structures according to this study’s participants. In another study, hospital reputation was one of the factors influencing patients’ choice of hospital in Iran [81]. In Cameroon, people can often go to a tertiary hospital without previous referral from a secondary or a primary hospital. As healthcare services in public institutions are out-of-pocket payments, these “good” hospitals will see an increase in their financing. Effective nonverbal communication with older patients goes a long way. It not only benefits individuals but hospitals and society in general. Therefore, nurses should strive to sustain effective nonverbal communication with older patients. 

### 3.4. Relationships between Concepts

In this model, all categories and subcategories are directly or indirectly interlinked. The category “*effective nonverbal communication*” is the core category in this model. It comprises the modalities of effective nonverbal communication and its purposes, which are directly linked. For instance, one or more modalities of nonverbal communication can be used to achieve one or more purposes of nonverbal communication in one interaction between a nurse and an older patient; an affective touch coupled with physical proximity can be used to win trust in older patients. The next category is the action and interaction strategies that need to be implemented to achieve effective nonverbal communication between nurses and older adults. This category is directly linked to the core category and intervening conditions. For instance, to support verbal communication, get messages across, and convey empathy or win older adults’ trust, nurses should be aware of their nonverbal behaviors, “being angels”, reduce negative stereotypes about older adults, and put yourself in the shoes of older patients. This shows the links between the purposes of effective nonverbal communication, the actions that should be taken by nurses, and the intervening conditions.

Figure 1 also shows that effective nonverbal communication between nurses and older patients rests on certain pillars that are interlinked and serve together as a solid structure. This means that effective nonverbal communication relies on nurses’ intrinsic factors, positive views of older adults, awareness of nonverbal communication, and nonverbal communication skills. Effective nonverbal communication also relies on older adults’ related factors such as their positive moods, their non-critical medical condition, and their financial situation. The diagram also shows that nurses’ effective nonverbal communication with older patients takes place within a specific context, which is the healthcare encounter. It also depends on the type of interaction between the nurse and the older patient. For instance, if the interaction is more task-related, affective nurses can use touch and sustained eye gaze to convey a positive emotion. The nonverbal communication that happens in a particular healthcare encounter and during a particular type of interaction is expected to yield positive results, such as older patients’ compliance with care and improved nursing care, thus leading to shorter stays in hospitals and the enhanced reputations of these hospitals.

## 4. Limitations

Although this model of effective nonverbal communication falls under transactional models of communication, it focuses more on the role of nurses; thus, one could argue that this model is linear. Moreover, the model acknowledges that older patients also have a role to play in effective nonverbal communication between them and nurses, but emphasizes nurses as shapers of the communication. A greater number of older patients could have enriched the study findings. However, as confirmed by Hall, Longhurst, and Higginson [82] and Lam et al. [83], it was difficult to conduct research with older adults because of the lack of trust in the researcher, lack of interest in the topic, the involvement of family members, and difficulties in obtaining consent. In addition, most of the older adults could speak neither French nor English. This can be seen as a limitation. Another limitation is that the observations were overt; therefore, the proposed model relies only on participants’ reports of what happened as well as interpretations of the observations made. Video recordings of interactions could have captured more details that might not have been captured by the researcher. The last limitation is that views from other healthcare workers, who also communicate nonverbally with older patients in the same settings, could have further strengthened the model.

## 5. Conclusions

This model adds to the body of knowledge on nonverbal communication between nurses and patients. It also answers the United Nations’ call on more data on older adults from low-and-middle-income countries. This model also provides a tool to help nurses communicate more effectively with older patients who mostly rely on nonverbal communication. The improved communication with older patients is expected to improve the quality of care rendered and the reputation of clinical settings. It is therefore recommended that the model is tested, evaluated, and refined for better outcomes.

## Figures and Tables

**Table 1:** Sociodemographic characteristics of the participants.

Participant	Age (Years)	Gender	Hospital	Position	Types of Nurses	Years of Experiences
P1	26–35	Female	Hospital 1	Staff nurse	Degree nurse	4
P2	46–55	Female	Hospital 2	Middle unit manager	Diploma nurse	32
P3	36–45	Female	Hospital 2	Staff nurse	Diploma nurse	23
P4	36–45	Male	Hospital 2	Staff nurse	Geriatric nurse	11
P5	46–55	Female	Hospital 2	Unit manager	Geriatric nurse	30
P6	46–55	Female	Hospital 2	Staff nurse	Diploma nurse	11
P7	36–45	Female	Hospital 2	Staff nurse	Diploma nurse	9
P8	26–35	Female	Hospital 2	Staff nurse	Geriatric nurse	3
P9	26–35	Female	Hospital 2	Staff nurse	Geriatric nurse	6
P10	26–35	Female	Hospital 2	Staff nurse	Nurse aid	14
P11	36–45	Female	Hospital 1	Staff nurse	Diploma nurse	13
P12	36–45	Female	Hospital 2	Staff nurse	Geriatric nurse	5
P13	26–35	Female	Hospital 1	Middle unit manager	Degree nurse	10
P14	18–25	Female	Hospital 1	Student nurse	Degree program	2nd year
P15	18–25	Female	Hospital 1	Student nurse	Diploma program	1st year
P16	26–35	Female	Hospital 2	Student nurse	Student nurse aid	1st year
P17	26–35	Female	Hospital 2	Student nurse	Student nurse aid	1st year
P18	78	Female	Hospital 1	N/A	N/A	N/A
P19	65	Male	Hospital 2	N/A	N/A	N/A
P20	64	Female	Hospital 2	N/A	N/A	N/A
P21	82	Female	Hospital 2	N/A	N/A	N/A
P22	67	Male	Hospital 2	N/A	N/A	N/A
P23	61	Male	Hospital 1	N/A	N/A	N/A
P24	92	Male	Hospital 2	N/A	N/A	N/A
P25	70	Female	Hospital 2	N/A	N/A	N/A

**Table 2:** Extracts of the contextual conditions forming the basis for developing the model.

Context and environment	Linguistically fragmented country	*… We have different mother tongues, for example, because we are in a diversity of languages here in Cameroon.* (P2, middle unit manager, diploma in nursing) *Many of those 60 years and older do not speak neither French nor English. It is often the foufouldé* (tribe in Cameroon), *the Makas* (tribe in Cameroon) *and all that. After school, you find yourself in a region where you perhaps speak French and your own mother tongue while the patients do not understand French nor your mother tongue. What do you do? You use nonverbal communication*. (P1, staff nurse, degree in nursing)
	Negative stereotypes on older adults	*In Cameroon, we believe that older people want to cling to life. They do not want to die. When they feel that they are about to die, they can exchange their life with younger lives to increase their lifespan. That is why younger people do not want to be close to a dying older person. It is like the famous video game Mario. When your energy level gets low, you should collect coins to increase your lifespan to continue to run. It is the same with older people*. (P23, older man, 61 years) *For example, in my village, there is this belief that if you administer care to an old patient that is almost dying, that patient can recover and transfer his death to you*. (P12, staff nurse, specialization in geriatric nursing)
	Inexistent long-term care facilities	*I think it would be different because here* (in the hospitals), *younger patients are mixed with older patients to young ones. Whereas there* (long-term care facilities), *you only care for older patients. I think it will help them better to be among themselves. Unfortunately, we don’t have homes in Cameroon yet.* (P11, staff nurse, diploma in nursing) *I think other structures should be created because it seems that in Cameroon, a geriatric unit is only functional here. With the ageing population, we need private structures for older patients because some families tell us that they wish there was a place where they could send their parents there for three months. Here in the hospital, you cannot keep a patient for three months because of the shortage of beds. If the government could develop structures for older people, it would be great*. (P7, staff nurse, diploma nurse)

**Table 3:** Extracts of the core phenomenon forming the basis for developing the model.

Core Phenomenon	Channel: Artifacts (use of objects)	*When you want to know the time, you can show them a watch. So, we can use objects to pass a message, depending on what we want to tell the person*. (P8, staff nurse, specialization in geriatric nursing) *You put a bottle of water in front of them, so that they understand that you are giving them water, and they will drink*. (P7, staff nurse, diploma in nursing)
	Channel: Haptics	*Yes, we touch the cheeks, we touch the hands. We greet, but touch his hand, so that he feels that we are here.* (P10, staff nurse, nurse aid) *Sometimes it takes a touch to make a change in them* (older patients). (P17, student nurse, 1st year) *When the person is sleeping, I touch him gently to wake him up.* (P12, staff nurse, specialization in geriatric nursing)
	Purpose: Support verbal communication	*You use hand gestures, facial expressions, eye gazes, all that to support what you are saying to him*. (P1, staff nurse, degree in nursing) *For me, nonverbal communication is anything we said or do without using words. For instance, it can be body language, smiling, frowning, a tap on a back, eye gazing, grumbling, and a lack of reaction when someone speaks to you*. (P13, middle unit manager, degree in nursing)
	Purpose: Build relationships with older patients	*As we said earlier, nonverbal communication is a form of exchange, a form of conversation with the older patients. The goal, I believe, is to establish a very good therapeutic relationship because we have to reassure the person, we put them at ease. I think nonverbal communication is to better establish a relationship with the older person and to encourage her to open up more.* (P4, staff nurse, specialization in geriatric nursing) *Often, nonverbal communication is to initiate the first contact in the relationship with older patients. Along the way, they notice that when the nurses come, they wave at them. So, even when the nurses forget to wave, the patients wave at the nurses. As time goes by, smiling with them, sitting on the bed close to them will help build the relationship.* (P7, staff nurse, diploma in nursing)

**Table 4:** Extracts of the outcomes forming the basis for developing the model.

Outcomes	Compliance with care	*We had patients who were not talking when they first arrived, they totally refused to eat, but as we spent time with them, reassuring them all the time, touching them, they started to give in. That particular patient ended up taking his medication and eating by his own. That made us happy.* (P8, staff nurse, specialization in geriatric nursing) *… Even if she doesn’t like a particular drug, she will take it to please you in return.* (P7, staff nurse, diploma in nursing)
	Older patients’ satisfaction	*When they smile with me or touch my hand, I am happy; I am pleased. I wish I could stay longer here*. (P18, older woman, 78 years) *So, the gestures play a lot because they bring joy in the unit, they bring joy to the family members as well as to the patients. The patients feel that we are willing to listen to them, that we are willing and are doing everything to help them. They are happy*. (P10, staff nurse, nurse aid)
	Nurse messages go through	*… When you gesture with them, they understand that you are giving them water for instance. They will drink it. Your message went through. Therefore, it* (nonverbal communication) *helps.* (P7, staff nurse, diploma in nursing) *Most of the times when they* (nurses) *use gestures, I understand what they mean.* (P25, older woman, 70 years) *Even if I speak in French and the patient does not understand, at least he feels that someone is speaking. Through my gestures, he understands what I mean. He interprets my gestures and understands, and my messages get through easily*. (P8, staff nurse, specialization in geriatric nursing)

## Data Availability

Not applicable.
